# Influence of Extracellular Vesicles Isolated From Osteoblasts of Patients With Cox-Arthrosis and/or Osteoporosis on Metabolism and Osteogenic Differentiation of BMSCs

**DOI:** 10.3389/fbioe.2020.615520

**Published:** 2020-12-23

**Authors:** Tanja Niedermair, Christoph Lukas, Shushan Li, Sabine Stöckl, Benjamin Craiovan, Christoph Brochhausen, Marianne Federlin, Marietta Herrmann, Susanne Grässel

**Affiliations:** ^1^Institute of Pathology, University of Regensburg, Regensburg, Germany; ^2^Department of Orthopaedic Surgery, Experimental Orthopaedics, Centre for Medical Biotechnology (ZMB/Biopark 1), University of Regensburg, Regensburg, Germany; ^3^Chair of Arthroplasty, Center for Orthopaedics and Trauma Surgery, University Hospital Giessen and Marburg GmbH, Marburg, Germany; ^4^Department of Conservative Dentistry and Periodontology, University Medical Center Regensburg, Regensburg, Germany; ^5^IInterdisciplinary Center for Clinical Research (IZKF), Group Tissue Regeneration in Musculoskeletal Diseases, University Hospital Wuerzburg and Bernhard-Heine-Center for Locomotion Research, University of Würzburg, Würzburg, Germany

**Keywords:** extracellular vesicles, mesenchymal stem cells, osteoblasts, osteoarthritis, osteoporosis, EVs, osteogenic differentiation

## Abstract

**Background:** Studies with extracellular vesicles (EVs), including exosomes, isolated from mesenchymal stem cells (MSC) indicate benefits for the treatment of musculoskeletal pathologies as osteoarthritis (OA) and osteoporosis (OP). However, little is known about intercellular effects of EVs derived from pathologically altered cells that might influence the outcome by counteracting effects from “healthy” MSC derived EVs. We hypothesize, that EVs isolated from osteoblasts of patients with hip OA (coxarthrosis/CA), osteoporosis (OP), or a combination of both (CA/OP) might negatively affect metabolism and osteogenic differentiation of bone-marrow derived (B)MSCs.

**Methods:** Osteoblasts, isolated from bone explants of CA, OP, and CA/OP patients, were compared regarding growth, viability, and osteogenic differentiation capacity. Structural features of bone explants were analyzed via μCT. EVs were isolated from supernatant of naïve BMSCs and CA, OP, and CA/OP osteoblasts (osteogenic culture for 35 days). BMSC cultures were stimulated with EVs and subsequently, cell metabolism, osteogenic marker gene expression, and osteogenic differentiation were analyzed.

**Results:** Trabecular bone structure was different between the three groups with lowest number and highest separation in the CA/OP group. Viability and Alizarin red staining increased over culture time in CA/OP osteoblasts whereas growth of osteoblasts was comparable. Alizarin red staining was by trend higher in CA compared to OP osteoblasts after 35 days and ALP activity was higher after 28 and 35 days. Stimulation of BMSC cultures with CA, OP, and CA/OP EVs did not affect proliferation but increased caspase 3/7-activity compared to unstimulated BMSCs. BMSC viability was reduced after stimulation with CA and CA/OP EVs compared to unstimulated BMSCs or stimulation with OP EVs. ALP gene expression and activity were reduced in BMSCs after stimulation with CA, OP, and CA/OP EVs. Stimulation of BMSCs with CA EVs reduced Alizarin Red staining by trend.

**Conclusion:** Stimulation of BMSCs with EVs isolated from CA, OP, and CA/OP osteoblasts had mostly catabolic effects on cell metabolism and osteogenic differentiation irrespective of donor pathology and reflect the impact of tissue microenvironment on cell metabolism. These catabolic effects are important for understanding differences in effects of EVs on target tissues/cells when harnessing them as therapeutic drugs.

## Introduction

The presence of extracellular vesicles, i.e., exosomes (further referred to as EVs, according to Théry et al., 2018), was initially demonstrated in studies with normal and neoplastic cell lines as exfoliation of membranous vesicles containing 5’-nucleotidase activity (Trams et al., 1981). At first, they were regarded as waste products, but recently they were attributed with a possible therapeutic potential (Edgar, 2016).

EVs are defined as intraluminal vesicles (ILVs) that are released into the extracellular milieu by the fusion of multivesicular bodies (MVBs) with the plasma membrane (Edgar, 2016). Their size varies between 30 and 150 nm and they are packed with a specific set of molecules, consisting of RNA, DNA, and proteins. Some of these molecules can be found commonly in EVs from different parent cells, whereas other molecules reflect the cytosolic and membranous content of the cell of origin (Valadi et al., 2007; Conde-Vancells et al., 2008; Simpson et al., 2008; Mathivanan et al., 2010; Sokolova et al., 2011; Wu et al., 2015). EVs can be incorporated again by the parent cell or can transmit their content by a paracrine or endocrine way from cell to cell, thereby affecting the target cell (Ratajczak et al., 2006; Valadi et al., 2007).

Various studies have reported the participation of EVs from bone cells and chondrocytes in physiological and pathophysiological processes related to bone and cartilage. EVs from osteoclast precursors were able to enhance osteoclast formation *in vitro*, whereas RANK-rich EVs from mature osteoclasts inhibited osteoclastogenesis (Huynh et al., 2016). Pro-osteogenic EVs have been shown to trigger osteogenic differentiation of naÏve mesenchymal stem cells (MSCs) *in vitro* and *in vivo* (Narayanan et al., 2016). In another study, Sun et al. demonstrated that miR-214 enriched EVs from osteoclasts can inhibit osteoblast activity *in vitro* (Sun et al., 2016). Further, EVs isolated from chondrocytes promoted chondrogenesis of progenitor cells and maintained cartilage stability (Chen et al., 2018).

Another aspect is the promising therapeutic effect regarding bone and cartilage regeneration that has been proposed for mesenchymal stem cell (MSC)-derived EVs. MSC have self-renewal capacities and can be isolated from various tissue sources, making them easily accessible. In addition, they are able to differentiate into multiple cells and tissues and have immunomodulatory properties (Caplan, 1991; Phinney and Prockop, 2007). They can be cultured in a short time period and expanded to an appropriate cell number, making them optimal for therapeutic use from the point of handling (Parekkadan and Milwid, 2010). However, adverse effects have been described in preclinical models and in clinical applications (Grässel and Lorenz, 2014). Usage of MSC derived EVs can reduce complications, e.g., graft vs. host reaction, and has several advantages as lower immunogenicity and the ability to cross biological barriers (Gowen et al., 2020). Several studies already demonstrated promising results regarding MSC-derived EVs in musculoskeletal pathologies such as osteoarthritis (OA) and osteoporosis (OP). The injection of EVs from induced pluripotent stem cell-derived MSCs or from synovial membrane-derived MSCs enhanced chondrocyte migration, proliferation, and cartilage repair in a collagenase-induced murine OA model (Zhu et al., 2017). Using the same model, Cosenza et al. (2017) reported a chondroprotective role and reduced osteophyte formation after injection of BMSC derived EVs into the mouse knee. In a rat-based study about disuse osteoporosis, injection of human umbilical cord mesenchymal stem cell (HUCMSC) derived EVs could prevent bone loss, demonstrated by μCt analysis and HE staining (Yang et al., 2020).

Hence, the use of MSC-derived EVs could be beneficial for the treatment of two of the most common age-related musculoskeletal pathologies: OA and OP. OA is classified as a degenerative joint disease, affecting the whole joint with all its tissue components. Functional and structural changes occur in multiple joint tissues and result in damage and loss of cartilage and the remodeling of subchondral bone resulting in pain as the most disabling symptom for the patient (Litwic et al., 2013; Grässel and Aszodi, 2019). OP is characterized by an imbalance of bone formation and resorption, thereby affecting the integration of bone density and quality. This finally results in compromised bone strength and an increased risk for micro traumata and fragility fractures (NIH Consensus Development Panel on Osteoporosis Prevention Diagnosis Therapy, 2001). Although the observation of coxarthrosis (CA) in combination with OP is not frequently observed, it can occur simultaneously in the same patient (Pogrund et al., 1982).

The use of MSC-derived EVs might improve and simplify current treatment strategies of OA and OP. EVs isolated from bone cells and chondrocytes are able to modulate physiological and pathophysiological processes related to bone remodeling and cartilage repair. Hence, EVs derived from pathologically altered cells in bone and cartilage might influence the outcome of treatments by counteracting effects from “healthy” MSC-derived EVs. To gain a better inside into this topic, this study aims to analyze if EVs isolated from osteoblasts of patients with hip OA (coxarthrosis/CA), OP or a combination of both (CA/OP) affect the metabolism and osteogenic differentiation capacity of BMSCs differently.

## Materials and Methods

This study was conducted in agreement with the ethics committee (MSCs: Ethikkommission, Nr. 14-101-0189; osteoblasts: 18-1109-101) and with patients’ written informed consent before undergoing surgery (email: ethikkommission@klinik.uni.regensburg.de).

### Isolation and Culture of Human BMSCs

Bone marrow aspirate (from femoral heads) was obtained from patients (male and female, *n* = 10, age 69 ± 9 years) undergoing hip replacement surgery due to coxarthrosis. Density gradient centrifugation to isolate BMSCs was used according to established protocols. Afterward, cells were expanded (passage/P 1–3) in growth medium (StemMACS MSC Expansion Medium, #130091680, Miltenyi Biotec, Bergisch Gladbach, Germany) supplemented with 0.2% MycoZap^TM^ Plus-PR (#VZA2022, Lonza, Basel, Switzerland) (Leyh et al., 2014; Wessely et al., 2019). Human MSC-associated markers CD73^+^, CD90^+^, CD105^+^, CD19^–^ and CD34^–^ were verified via flow cytometry. Cells in P1–3 were stored in liquid N_2_ until further use.

For the experiments, BMSCs in P2 were thawed and seeded in growth medium until 80% confluency. Cells were harvested and seeded in growth medium in 96-well plates (triplicates; 5,000 cells/well) for BrdU, Caspase 3/7, and WST-1 assays. For Alizarin and ALP-assays, cells were cultured for 28 days in osteogenic medium in 12-well plates (duplicates; 10,000 cells/well). For gene expression analysis (RT-qPCR, duplicates), cells were cultured for 13 days in 6-well plates in osteogenic medium (30,000 cells/well). Cells of different donors were not pooled for the experiments.

To isolate EVs, BMSCs of P3 were thawed and cultured in growth medium until 85–90% confluency. Afterward, cells were washed with PBS and cultured in collection medium (CM) for 48 h [CM: α-MEM, #M8042, Sigma-Aldrich, Steinheim, Germany, + 10% exosome depleted FCS (self-processed via ultracentrifugation, FCS^*depl.*–*uc*^) (#F7524, + 1% P/S-#A5955, Sigma-Aldrich, Steinheim, Germany)]. CM was collected and stored at −80°C for further processing, cells were passaged and the procedure was repeated until P6 (Supplementary Figure 1A).

### Isolation and Culture of Human Osteoblast-Like Cells

**FIGURE 1:**
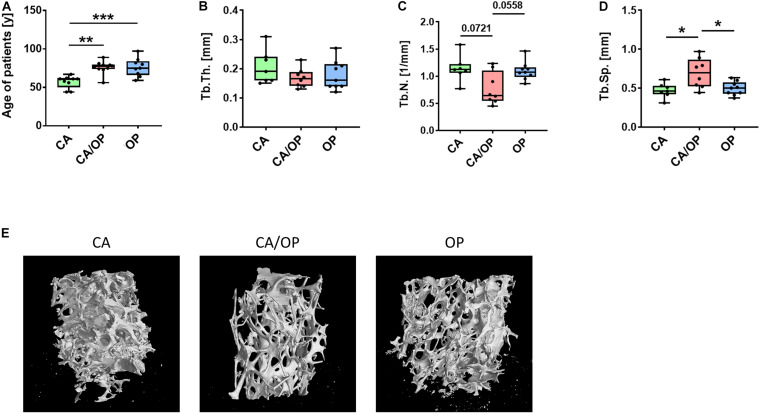
Patient characteristics and μCT analysis of bone biopsies obtained from CA, CA/OP, and OP patients after hip replacement surgery. **(A)** Age of patients with CA, CA/OP, and OP at time point of operation. *N* = 9 (CA), 10 (CA/OP), 9 (OP); Y = years. **(B)** μCT analysis of bone explants from trabecular bone—trabecular thickness (Tb.Th.). *N* = 7 (CA), 8 (CA/OP), 9 (OP). **(C)** μCT analysis of bone explants from trabecular bone—trabecular number (Tb.N.). *N* = 7 (CA), 8 (CA/OP), 9 (OP). **(D)** μCT analysis of bone explants from trabecular bone—trabecular separation (Tb.Sp.). *N* = 7 (CA), 8 (CA/OP), 9 (OP). **(E)** Representative 3D-images of bone explants of CA, CA/OP, and OP patients after μCT analysis. Scanning was performed with a voxel size of 12 μm.

**FIGURE 2:**
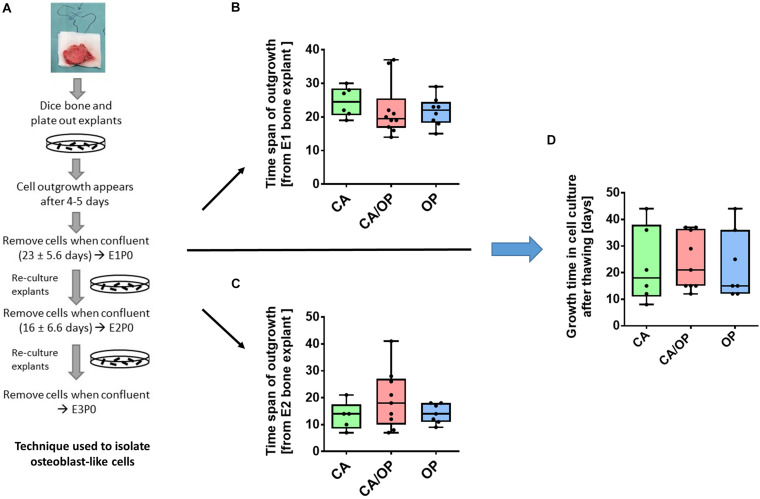
Isolation of osteoblast-like cells from bone explants of patients with CA, CA/OP, and OP. **(A)** Isolation process demonstrating cell outgrowth of osteoblast-like cells expressing osteoblastic characteristics from bone explants in two consecutive phases of explant (E)1, E2, and E3 cultures. Cells were either frozen immediately in passage 0 (P0) or frozen after additional passaging of the cells until P3 (modified from Dillon et al., 2012). **(B)** Time span of outgrowth of cells from E1 bone explants until confluency. *N* = 6–10. **(C)** Time span of outgrowth of cells from E2 bone explants until confluency. *N* = 5–9. **(D)** Growth time of cells in cell culture from time point of seeding the cells after thawing until confluency. *N* = 6–9.

Osteoblast-like cells were isolated from bone explants (bone disc of the femoral neck) of female patients with coxarthrosis (CA, *n* = 9), osteoporosis (OP, *n* = 9) or a combination of both (CA/OP, *n* = 10) undergoing hip-replacement surgery or after femoral neck-fracture (Age range of female donors: Figure 1A). Bone was cut into small explants, washed several times with PBS and placed in T25 culture flasks in growth medium (DMEM low glucose, #31885-023, Gibco Life Technologies, Darmstadt, Germany) supplemented with 10% FCS, 1% P/S and 100 μM ascorbic acid-2-phosphate (#F7524; #A5955; #A8960, all Sigma-Aldrich, Steinheim, Germany). After 4–5 days, cells start to migrate out of the bone explants (E1/P0, Figure 2A; Dillon et al., 2012). Cells were cultured until confluency, harvested and stored under frozen conditions. Process was repeated with bone explants as E2; cells were stored as E2/P0 and process was repeated one time as E3 (E3/P0). Time span of outgrowth was noted in each explant culture. Cells of E1–E3/P1–3 were used for the experiments.

For the experiments, osteoblast-like cells of E1, 2, and 3 (cells from different donors were not pooled) from CA, OP, and CA/OP patients were thawed and cultured in growth medium until confluency. Time span until confluency was noted. Cells were harvested and seeded in osteogenic medium for 14, 21, 28, and 35 days. Cells were seeded in 96-well plates (5,000 cells/well, triplicates) for the WST-1-assay, in 12-well plates (20,000 cells/well, duplicates) for Alizarin Red quantification and alkaline phosphatase (ALP) assay and in 6-well plates (40,000 cells/well) for gene expression analysis (RT-PCR, duplicates).

To isolate EVs, osteoblast-like cells were thawed, expanded in growth medium until P2–3 and cultured until 80–90% confluency. Medium was then changed to osteogenic medium [α-MEM, (#M8042, Sigma-Aldrich, Steinheim, Germany) + 10% FCS, 1% P/S, 4 mM GlutaMAX^TM^-I (100×, #35050038, Gibco, Paisley, United Kingdom), 10 μM ascorbic acid-2-phosphate, 10 mM β-glycerophosphate (#G9891, Sigma-Aldrich, Steinheim, Germany) and 100 nM dexamethasone (#D4902, Sigma-Aldrich, Steinheim, Germany)] in order to induce osteogenic differentiation of the cells for 35 days. The term “osteoblasts” is used for osteoblast-like cells in osteogenic culture, to clearly distinguish between cells directly after isolation and cells in osteogenic culture. On day 26, cells were washed with PBS and cultured in osteogenic CM (osteogenic medium + 10% FCS^*depl.*–*uc*^) until day 28, then CM was collected (day 28) and process was repeated until day 35. CM was stored at −80°C for further processing (Supplementary Figure 1A).

### EV-Depleted FCS

EV-depleted FCS (FCS^*depl.*–*uc*^) was prepared according to Lässer et al. (2012). Undiluted FCS was centrifuged at 120,000×*g* overnight (minimum 16 h) (Optima L-90 K ultracentrifuge, SW32-Ti rotor, Beckman Coulter, Brea, United States) at 4°C and EV-depleted supernatant (FCS^*depl.*–*uc*^) was collected by pipetting. Special care was taken to not disturb the pellet at the bottom of the tube (Polypropylene Centrifuge Tubes; Beckman Coulter, Brea, United States), therefore up to 1 cm liquid was left. The collected FCS^*depl.*–*uc*^ was stored in aliquots at −20°C. Medium for EV collection and stimulation was supplemented always with 10% FCS^*depl.*–*uc*^.

### EV Isolation

Isolation of EVs from MSC and osteoblast CM was performed by ultracentrifugation according to a modified protocol from Théry et al. (2006). In brief, MSC and osteoblast CM was centrifuged at 300×*g* (Sigma 3K30, Sigma Laborzentrifugen GmbH, Osterode a. Harz, Germany) for 10 min at 4°C to remove cells. Supernatant was transferred to a new falcon tube and centrifuged at 2,000×*g* for 10 min at 4°C to remove dead cells. Afterward, supernatant was transferred to a new tube and cleaned from debris (10,000×*g*, 30 min, 4°C). Next, supernatant was filtered (Filtropur S plus 0.2 μm; Sarstedt, Nümbrecht, Germany) into an ultracentrifugation tube (Polypropylene Centrifuge Tubes; Beckman Coulter, Brea, United States) and centrifuged once at 120,000×*g* for 70 min at 4°C (Optima L-90 K ultracentrifuge, SW32-Ti rotor, Beckman Coulter, Brea, United States). Then, supernatant was removed carefully and the pellet was washed with PBS and centrifuged again (120,000×*g* for 70 min at 4°C). PBS was discarded except for 200–300 μl residual volume for resuspending the EV pellet. Protein concentration was measured using BCA Protein Assay Kit Pierce (Thermo Scientific, Rockford, United States). Osteoblast-derived EV from each patient (days 28–35) were pooled before last washing steps to increase EV yield. Finally, EVs derived from naïve BMSCs and CA, OP, and CA/OP osteoblasts were stored until usage in aliquots at −80°C (Supplementary Figure 1B).

### Western Blot Analysis of EV Markers CD9 and CD81

1 × 10^6^ BMSCs were lysed by ultrasound treatment in 150 μl heat-hot lysis buffer (10 mM TRIS-HCl, Carl Roth, Karlsruhe, Germany; 1%SDS, Carl Roth, Karlsruhe, Germany). Protein concentration was determined using BCA-assay (#23227, Thermo Scientific, Rockford, United States). Five microgram of BMSC lysate (lane 2), 8.2 μg (lanes 3–6) of purified EVs and 10 μl of undiluted FCS^*depl.*–*uc*^ (lane 7) were separated by SDS-PAGE. Then, proteins were blotted onto a PVDF membrane (0.2 μm; Roche, Mannheim, Germany), stained with Ponceau red solution (#09189-1L-F, Sigma-Aldrich, Steinheim, Germany, see Supplementary Figure 3), blocked with 5% BSA (Carl Roth, Karlsruhe, Germany) in 0.1% Tween Tris Buffered Saline (T-TBS) and consecutively incubated with the following primary antibodies in 5%BSA/T-TBS overnight at 4°C: mouse monoclonal anti-CD9 (Ts9, #10626D) and mouse monoclonal anti-CD81 (M38, #10630D) (all 1:1,000; Thermo Fisher Scientific, Rockford, United States). After three washing steps, 10 min each, membranes were incubated with horseradish peroxidase coupled secondary antibody (1:20,000; Jackson Immuno Research, West Grove, United States) for 1 h at room temperature (RT). Proteins were visualized using SuperSignal West Femto Maximum Sensitivity Substrate (#34095; Thermo Fisher Scientific, Rockford, United States) according to manufacturers’ protocol. Images were prepared using Photoshop 7.0 (Adobe).

### EV Staining With PKH-26

EVs and PBS (control) aliquots were stained with the PKH26 Red Fluorescent Cell Linker Mini Kit for General Cell Membrane Labeling (#PKH26GL-1KT, Sigma-Aldrich, Steinheim, Germany) according to a slightly modified manufacturer’s protocol. PBS was used to control for false positive signals (Wallace et al., 2008; Lai et al., 2015). Diluent C was added to EVs and PBS up to 1 ml volume. Then, 6 μl PKH-26 staining solution was added, mixed, and incubated for 5 min at RT. To stop the dyeing process, 2 ml of 5% BSA in PBS were added, solutions were transferred to ultracentrifugation tubes, filled up with PBS and centrifuged at 120,000×*g* for 70 min at 4°C. The supernatant was discharged, the pellet was resuspended in PBS and stored on ice. BMSCs were expanded as described (see section “Isolation and Culture of Human BMSCs”), seeded on chamber slides (Corning, Big Flats, United States, 10,000 cells/chamber) and cultured in growth medium for 48 h. Cells were washed with PBS and growth medium with FCS^*depl.*–*uc*^ and the pre-stained EVs or PBS were added for 24 h. Nuclei were counterstained with DAPI (Molecular Probes, Eugene, United States) and staining was analyzed using a fluorescence microscope (Eclipse TE2000-U; Nikon, Tokyo, Japan).

### Transmission Electron Microscopy (TEM) Analysis of EVs

Freshly isolated naïve BMSC EVs were resuspended in cold PBS. For negative staining, 20 μl were added on a parafilm and a formvar (polyvinyl formal)-carbon coated 400 copper mesh grid (#G400-CU; Science Services, Munich, Germany) was placed on top of the fluid for 10 min at RT. The grid was incubated with 2% phosphotungstic acid (#19500; Science Services, Munich, Germany) for 1 min and dried at RT for 10 min. EVs were investigated with an acceleration voltage of 100 kV and 100,000× magnification using a Leo 912 AB (Carl Zeiss, Oberkochen, Germany).

### Scanning Electron Microscopy (SEM) Analysis of EVs

EVs from naïve BMSCs were dehydrated through a graded series of alcohol (1 ml of 30, 50, 70, 80, 90, and 96%) with centrifugation of 2 × 10 min between each washing step. EVs were washed with absolute EtOH, centrifuged at 60,000×*g* for 10 min and supernatant was discharged. The pellet was resuspended in 50 μl absolute EtOH and incubated for 2 h on a 13 mm tissue culture coverslip (#83.1840.002; Sarstedt, Nümbrecht, Germany). Then, 200 μl absolute EtOH was added and the sample was processed in a critical point dryer and sputtered with platinum. Imaging was performed with an acceleration voltage of 3 kV, spot size of 2, 5–6 mm working distance and an aperture of 30 μm using a FEI Quanta 400 FEG (FEI, Frankfurt a. Main, Germany).

### Stimulation of BMSCs With EVs

For BrdU, Caspase 3/7-activity, and WST-1 assay, BMSCs were stimulated with 10 μg/ml of naïve BMSC, CA, OP, or CA/OP EVs in growth medium. For BrdU and Caspase 3/7-activity assays, cells were cultured for 2 days and subsequently stimulated for 24 h. For the WST-1 assay, cells were cultured 2 days and stimulated for 48 h. For Alizarin Red-, ALP-, and RT-qPCR analysis, BMSCs were stimulated with 10 μg/ml of naïve BMSC, CA, OP, or CA/OP EVs in osteogenic medium. For RT-qPCR analysis, cells were cultured for 13 days, then stimulated for 24 h, and for Alizarin Red- and ALP-analysis, cells were cultured for a total of 21 days. EVs were added during the last 7 days with every medium exchange (3 times). PBS was used for unstimulated BMSC cultures as control (Ø, no EVs) (Supplementary Figure 2).

### BrdU Proliferation Assay

To analyze cell proliferation, BMSCs were cultured in 96-well plates for 2 days. Then, cells were stimulated with EVs for 24 h, simultaneously BrdU reagent was added. Cell proliferation ELISA (colorimetric BrdU, Roche, Mannheim, Germany) was further conducted according to manufacturer’s protocol. Results were calculated as percentage of No EV-controls.

### Caspase-3/7 Assay

BMSCs were cultured for 2 days, followed by EV stimulation for 24 h. Afterward, Caspase 3/7-activity was determined as an indicator for apoptosis using the Apo-ONE^®^ Homogeneous Caspase-3/7 assay (Promega, Madison, United States) according to manufacturer’s specifications. Results were calculated as percentage of No EV-controls.

### Viability Assay (WST-1 Assay)

For comparison of CA, OP, and CA/OP osteoblasts, cells were cultured for 14, 21, 28, and 35 days. For experiments, BMSCs were cultured for 2 days, then, cells were stimulated with EVs for additional 48 h. At respective time points, WST-1 reagent was added (Roche, Mannheim, Germany; 10 μl/well) and absorbance was measured after 30 min (BMSC cultures) or 1 h (osteoblast cultures). For BMSC cultures, results were calculated as percentage of No EV-control.

### RNA Isolation and Real-Time PCR (RT-PCR)

RNA from BMSCs was isolated after 14 days in osteogenic medium and stimulation with EVs for the last 24 h of culture time. RNA from osteoblasts of CA, OP, and CA/OP patients was isolated after 28 and 35 days in osteogenic medium to analyze gene expression profile at time points of EV collection. Cells were cultured in 6-well plates and lysed at respective time points with 100 μl of β-mercaptoethanol containing lysis buffer. RNA was further isolated using Absolutely RNA Microprep Kit (Agilent Technologies, Cedar Creek, United States) according to manufacturer’s instructions, including DNAse treatment, with minor modifications during RNA elution steps (3 min incubation of elution buffer and 5 min centrifugation). RNA-concentration was quantified (NanoDrop 2000; Peqlab, Erlangen, Germany) and cDNA was prepared using AffinityScript QPCR cDNA Synthesis Kit (Agilent Technologies, Cedar Creek, United States) as recommended by manufacturer’s protocol. PCR was performed in duplicates using the Brilliant III Ultra-Fast SYBR Green QPCR Master Mix with an Agilent PCR-System (Agilent Technologies, Cedar Creek, United States). The following primers were used (5’–3’): *GAPDH* (house keeper): fwd, CTGACTTCAACAGCGACACC, rev, CCCTG TTGCTGTAGCCAAAT; *ALP*: fwd, CCTCCTCGGAAGACA CTCTG, rev, AGACTGCGCCTGGTAGTTGT; *BGLAP*: fwd, GTGCAGAGTCCAGCAAAGGT, rev, TCAGCCAACTCGTC ACAGTC; *COL1A1*: fwd, ACGTCCTGGTGAAGTTGGTC, rev, ACCAGGGAAGCCTCTCTCTC; *Runx2*: fwd, CGGAATG CCTCTGCTGTTATG, rev, GCTTCTGTCTGTGCCTTCTG. Results of osteoblast cultures were calculated by the ΔCT method in relation to GAPDH expression. Results of BMSC cultures were further calculated as x-fold change to calibrator (= ΔΔCt method; calibrator = No EV-control).

### ALP Enzyme Activity

Intracellular ALP enzyme activity was analyzed as described previously (Niedermair et al., 2018, 2020). Briefly, osteoblasts were seeded in 12-well plates and cultured in osteogenic medium for 14, 21, 28, and 35 days. BMSCs were seeded likewise and cultured for 28 days in osteogenic medium. BMSCs were stimulated with EVs from days 21 to 28 (3× EV supplemented medium exchange). QuantiChrom Alkaline Phosphatase Assay Kit (BioAssay Systems, Hayward, United States) was used according to manufacturer’s manual to quantify intracellular ALP enzyme activity. Results were calculated as μmol/l^∗^min, results for BMSC cultures were further calculated as percentage of “No EV-control.”

### Alizarin Red Staining and Photometric Analysis

Calcium deposition was measured by Alizarin Red staining and subsequent quantification. Osteoblasts were cultured for 14, 21, 28, and 35 days in 12-well plates in osteogenic medium. BMSCs were cultured likewise for 28 days and stimulated from days 21 to 28 with EVs (3× EV supplemented medium exchange). At specific time points, cells were washed with PBS and fixed with 1% glutaraldehyde for 15 min at RT. After a washing step with PBS (pH = 4.2), cells were incubated for 20 min at 37°C with Alizarin-S staining solution (1%, #0348.3, Carl Roth, Karlsruhe, Germany). Cells were washed again 2× with PBS and photometric quantification of Alizarin Red staining was performed according to a protocol by Gregory et al. (2004). Briefly, PBS was discharged, cells and matrix were detached with 200 μl of 10% acetic acid for 30 min and transferred to a new cup using a cell scraper. Cups were vortexed, incubated at 85°C for 10 min, cooled on ice for 5 min and centrifuged at 20,000×*g* for 15 min at 4°C. Supernatant was transferred and pH was adjusted to 4.4–4.5. A buffer of 8 parts 10% acetic acid and 3 parts 10% ammonium hydroxide was prepared for the standard curve (2, 1, 0.5, 0.25, 0.125, 0.0625, and 0.0312 mM). Fifty microliter of sample and standard were measured in a 96-well plate at 405 nm. Results were calculated as mM Alizarin Red. For BMSC cultures, results were further calculated as percentage of No EV-control.

### μCT Analysis of Bone Samples

Trabecular bone samples (length/width/height about 0.5–0.7 cm) were cut out directly from the bone pieces after receiving the samples and stored frozen at −80°C until μCT analysis. Samples were scanned in a micro-computed tomography system with a voxel size of 12 μm (Röntgenprüfsystem v| tome| x s 240 Research/Edition V2.5, GE Sensing and Inspection Technologies GmbH, Wunstorf, Germany, DFG number: INST 102/11-1 FUGG). Following parameters were used for scanning: X-ray tube was operated at 45 kV and 260 μA; integration time of 333 ms and 1,500 images/360°. Automatic geometry calibration without using further filters was used for reconstruction of the data. Bone microarchitecture was analyzed with the Bruker CtAN Software (Bruker Corporation, Billerica, MA, United States).

### Statistical Analysis

GraphPad Prism 8 (GraphPad, San Diego, United States) was used for statistical analysis and graph preparation. Data in box plots are expressed as median + min/max. Differences between the groups were analyzed using two-tailed Mann-Whitney U-test and Wilcoxon signed-rank test was applied when No EV-control was set to 100%. *P* < 0.05 were considered as significant.

## Results

### Patients Characteristics and μCT Analysis of Bone Explants From CA, OP, and CA/OP Patients

Mean age of CA patients (57.11 ± 8.0 years) was significantly lower compared to mean age of CA/OP (76.10 ± 8.4 years) and OP (76.11 ± 11.7 years) patients (Figure 1A). μCT analysis was performed with trabecular bone samples of a disc, cut out from the femoral neck during hip replacement surgery. No difference in trabecular thickness (Tb.Th.; Figure 1B) was detected, but trabecular number (Tb.N.; Figure 1C) was lower by trend and trabecular separation (Tb.Sp.; Figure 1D) was higher in bone samples of CA/OP compared to CA and OP patients. Representative μCT images of bone explants of all groups are shown under Figure 1E.

### Growth Characteristics of Osteoblasts Isolated From CA, OP, and CA/OP Patients

Osteoblast-like cells were obtained from patient bone explants during *in vitro* explant cultures E1, E2, and E3. Processing of bone explant cultures as described by Dillon et al. (2012) is schematically presented in Figure 2A. No difference was observed regarding time span of cell outgrowth from bone explants until confluency between CA, CA/OP, and OP osteoblast-like cells in E1 or E2 explant cultures (Figures 2B,C). In addition, growth time until confluency in culture after thawing was comparable in all groups (Figure 2D).

### Viability and Osteogenic Differentiation Capacity of CA, CA/OP, and OP Osteoblasts

**FIGURE 3:**
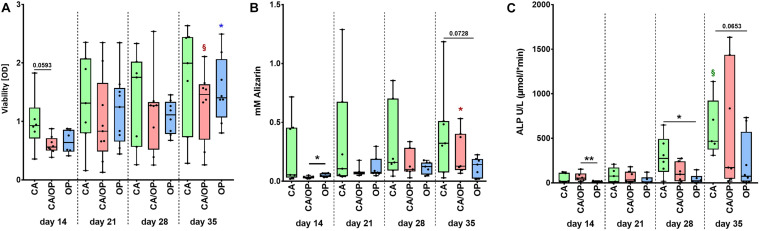
Characterization of viability and osteogenic capacity of osteoblasts isolated from patients with CA, CA/OP, and OP. **(A)** Comparison of osteoblast viability [optical density = OD]. *P*-values over bars represent differences between groups (CA; CA/OP; OP). ^§^ (Red) = *p* = 0.0781, represents tendency compared to the CA/OP group at day 14. *(blue) = *p* ≤ 0.05, represents difference compared to the OP group at day 14. *N* = 6–9. **(B)** Matrix formation capacity was compared using Alizarin Red staining. Graph shows concentration of Alizarin Red in mM. *P*-values over bars or *(= *p* ≤ 0.05) represent differences between groups (CA; CA/OP; OP). *(red) = *p* ≤ 0.05, represents difference compared to the CA/OP group at day 14. *N* = 7–8. **(C)** Bone formation ability was compared by assaying intracellular ALP enzyme activity (U/L). *P*-values over bars or *(= *p* ≤ 0.05)/**(= *p* ≤ 0.01) represent differences between groups. ^§^ (Green) = *p* = 0.0625, represents tendency compared to the CA/OP group at day 14. (CA; CA/OP; OP). *N* = 6–8.

Before collection of culture supernatant for EV isolation, we compared viability, calcium deposition ability (Alizarin Red quantification) and ALP enzyme activity between CA, CA/OP, and OP osteoblasts throughout the osteogenic differentiation time line. Viability was by trend higher in CA compared to CA/OP osteoblasts after 14 days in osteogenic medium. No further differences were observed between the groups but viability increased by trend during osteogenic differentiation (days 14–35) for CA/OP and significantly for OP osteoblasts (Figure 3A). Calcium deposition was higher in OP compared to CA/OP osteoblasts after 14 days and in CA compared to OP osteoblasts (trend) after 35 days in osteogenic medium. Calcium deposition increased significantly from days 14 to 35 in CA/OP osteoblast cultures (Figure 3B). Intracellular ALP enzyme activity was higher in CA/OP compared to OP osteoblasts after 14 days in osteogenic medium. During later osteogenic differentiation, after 28 and 35 days, ALP activity was higher in CA compared to OP osteoblasts. ALP enzyme activity increased by trend in CA osteoblasts during osteogenic differentiation (days 14–35) (Figure 3C).

### Expression of Osteogenic Marker Genes in CA, CA/OP, and OP Osteoblasts

**FIGURE 4:**
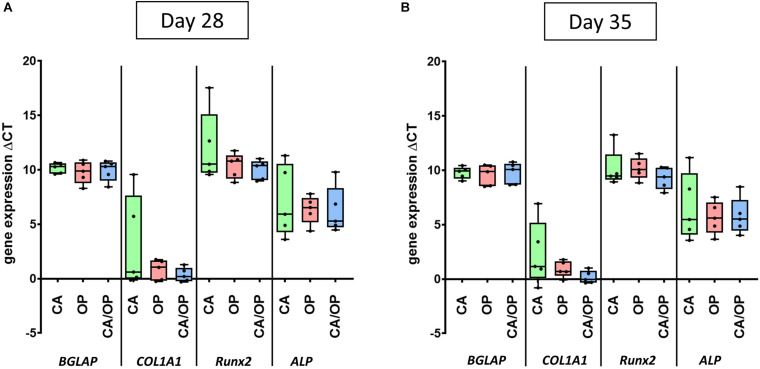
Gene expression of *BGLAP, COL1A1, Runx2*, and *ALP* in CA, OP, and CA/OP osteoblasts after 28 **(A)** and 35 **(B)** days in osteogenic culture medium. These time points were chosen according to time points for EV isolation from the culture supernatants of osteogenic cultured CA, OP, and CA/OP osteoblasts. Results were calculated by the ΔCt method. *N* = 4.

Culture supernatant for EV/exosome isolation was collected during late osteogenic differentiation, therefore expression of osteogenic marker genes *BGLAP, COL1A1, RUNX2*, and *ALP* was compared between CA, CA/OP, and OP osteoblasts after 28 and 35 days. Results of the RT-qPCR analysis were calculated as ΔCT-values in relation to *GAPDH* expression indicating that a lower ΔCT-value would represent higher gene expression levels, whereas a higher ΔCT-value demonstrates lower gene expression levels. Our data revealed no differences regarding gene expression of *BGLAP, COL1A1, Runx2*, and *ALP* between CA, CA/OP, and OP osteoblasts after 28 and 35 days in osteogenic medium (Figures 4A,B).

### SEM and TEM Evaluation of Isolated EVs From Naïve BMSCs

**FIGURE 5:**
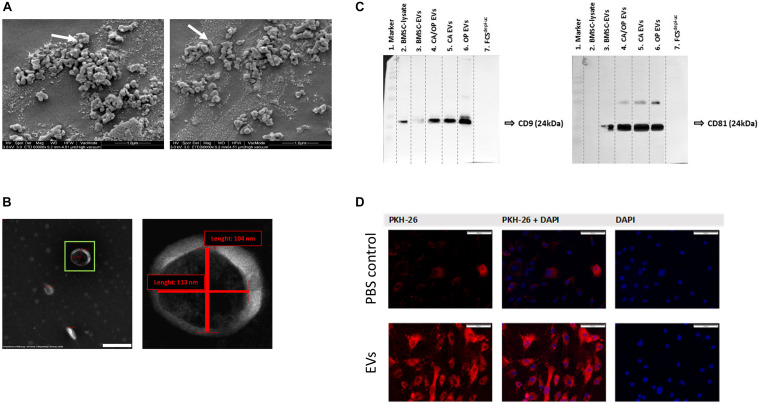
EV characterization and validation. **(A)** Representative SEM pictures of BMSC EVs. White arrows label EVs with 97 nm (left image) and 115 nm (right image) diameter. Magnification: 60,000×. **(B)** Representative TEM pictures of BMSC EVs. Scale bar left image = 200 nm, magnification: 100,000×. Right image shows magnification of the EV in the green box. The size of objects was determined (red lines). Vertical line = 104 nm, horizontal line = 113 nm. **(C)** Western Blot analysis of EV specific surface markers CD9 (left image) and CD81 (right image) in BMSC, CA, CA/OP, and OP EVs and in the EV-depleted FCS^*depl*– *uc*^. Lane 1 = MW ladder, Lane 2 = 5 μg; Lanes 3–6 = 8.2 μg; Lane 7 = 10 μl; Exposure time: left image = 3 min, right image = 10 min (Pierce femto Kit). For respective Ponceau Red images, see Supplementary Figure 3. **(D)** Test for EV uptake of BMSC-derived EVs into cultured BMSCs using PKH-26 (red) stained EVs (lower panel). PBS solution + PKH-26 stain was used as negative control (upper panel). Nuclei were counterstained with DAPI. Scale bar 100 μm.

BMSC-derived EVs were characterized using SEM. Here, numerous spherical particles were revealed having a round and uniform morphology. The distribution of size was unimodal with an average range of about 100 nm (Figure 5A, 97 nm—left image, 115 nm—right image). To further validate the isolated particles as EVs, TEM analysis was performed. We detected particles with the typical cup-shaped morphology as a characteristic EV feature, including exosomes. The depicted structure in Figure 5B (left image—green box) had a length about 113 nm horizontal and about 104 nm vertical (right image).

### Western Blot Analysis for Exosome Markers CD9 and CD81

Western blotting was performed to verify the presence of EV membrane markers CD9 and CD81. EVs isolated from BMSCs, CA, OP, and CA/OP osteoblasts were analyzed and whole BMSC-lysates were used as positive control. In addition, FCS^*depl.*–*uc*^ was analyzed, to control for absence of EV markers. CD9 positive bands were detected at a size of about 24 kDa in the BMSC lysate, BMSC EVs as well as CA, CA/OP, and OP EVs (Figure 5C, left image). Positive bands for CD81 were detected at 25 kDA in BMSC, CA, CA/OP, and OP EVs, but not in the BMSC cell lysate (Figure 5C right image). No bands were detected in undiluted FCS^*depl.*–*uc*^. Supplementary Figure 3 shows respective images of Ponceau red stained membranes.

### EV Uptake

PKH-26 staining was performed to analyze the uptake of EVs by BMSCs. The upper row of Figure 5D shows confocal fluorescent microscopy data for PBS instead of PKH-26 stained EVs as control for false positive staining (PKH-26 in left image, DAPI in right image, overlay in middle image). Some unspecific dyed micro-particles were observed in the negative control, that had been internalized by BMSCs. In contrast, intense red staining was recorded intracellularly after incubation of BMSCs with the PKH-26 labeled EVs (Figure 5D, lower row). Red structures were localized to intracellular compartments as a result of internalization of PKH-26 stained EVs.

### Effects of BMSC-, CA-, CA/ OP-, and OP-Derived EVs on BMSC Metabolism

**FIGURE 6:**
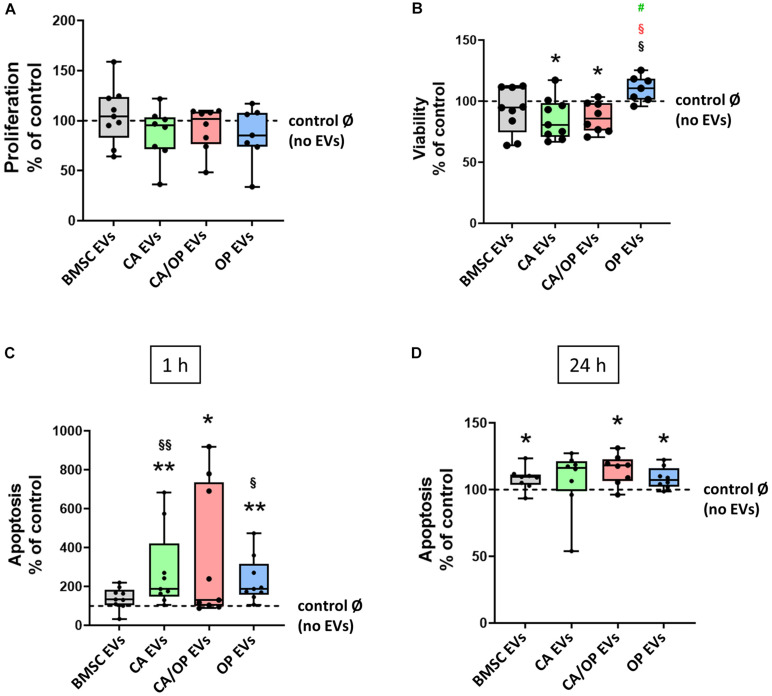
Influence of EVs isolated from BMSCs and CA, CA/OP, and OP osteoblasts on BMSC metabolism. **(A)** Proliferation of BMSCs after cultivation for 2 days followed by stimulation for 24 h with EVs isolated from BMSCs and CA, CA/OP, and OP osteoblasts. *N* = 9. **(B)** Viability (WST-1 assay) of BMSCs after cultivation for 2 days followed by stimulation for 48 h with EVs isolated from BMSCs and CA, CA/OP, and OP osteoblasts. *N* = 9. **(C,D)** Apoptosis rate (Caspase 3/7 activity assay) of BMSCs after cultivation for 2 days followed by stimulation for 24 h with EVs isolated from BMSCs and CA, CA/OP, and OP osteoblasts. Fluorescence was measured 1 h and 24 h after addition of Caspase 3/7-assay buffer. *N* = 9. Results were calculated as percentage to the unstimulated control (BMSCs without EV stimulation/no EVs/Ø = 100%, shown by the dotted line). */**Significant differences to control Ø with *p* ≤ 0.05/0.01; §/§§Significant differences to BMSC EVs (EVs) with *p* ≤ 0.05/0.01. ^§^ (red) = significant differences to CA/OP EVs with *p* ≤ 0.05. ^#^(green) = tendency compared to CA EVs.

Proliferation, viability and Caspase 3/7 activity were analyzed to test the impact of naïve BMSC-, CA-, CA/ OP-, and OP-derived EVs on the metabolism of BMSCs. Positive effects had been reported in the literature for BMSC EVs on these parameters. Therefore, we used naïve BMSC EVs to control for CA, OP, and CA/OP EV-mediated effects. In none of the groups, effects on cell proliferation were detected (Figure 6A). Cell viability was significantly decreased after stimulation with CA and CA/OP EVs compared to the No EV-control. Stimulation with OP EVs increased viability of BMSCs compared to the other three groups (Figure 6B). Apoptosis rate of BMSCs treated with CA and OP EVs was highly increased compared to cells stimulated with naïve BMSC EVs and compared to the “No EV-control.” Higher apoptosis rate was recorded in CA/OP EV-treated BMSCs compared to the No EV-control. Caspase 3/7 activity was not affected in BMSC EV-treated group (Figure 6C, 1 h assay time point). Comparable results were obtained for the 24 h assay time point (Figure 6D). Stimulation of BMSCs with CA/OP, OP, and naïve BMSC EVs increased apoptosis rate compared to the “No EV-control,” whereas no difference to the “No EV-control” was observed after 24 h for the CA EV-treated BMSC group.

### Effects of BMSC-, CA-, CA/ OP-, and OP-Derived EVs on Expression of Osteogenic Marker Genes in BMSCs

**FIGURE 7:**
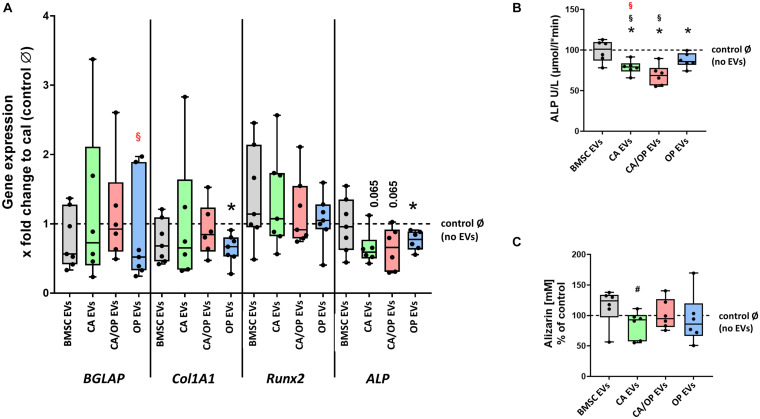
Influence of stimulation with EV from BMSCs and CA, CA/OP, and OP osteoblasts on the expression of osteogenic marker genes in BMSCs and their osteogenic differentiation ability. **(A)** Gene expression of *BGLAP, COL1A1, RUNX2*, and *ALP* was analyzed after cultivation for 13 days followed by a 24 h stimulation of BMSCs with EVs isolated from BMSCs and CA, CA/OP, and OP osteoblasts. Calibrator (= no EVs/Ø control) was set to 1 and results were calibrated as x-fold change to calibrator (= ΔΔCt method). *N* = 7. **(B)** ALP enzyme activity as a marker for bone formation activity in BMSCs after cultivation for 21 days followed by stimulation during the last 7 days (EV addition 3× with medium exchange) with EVs isolated from BMSCs and CA, CA/OP, and OP osteoblasts. *N* = 7. **(C)** Alizarin Red incorporation into the extracellular matrix of BMSCs after cultivation for 21 days followed by stimulation during the last 7 days (EV addition 3× with medium exchange) with EVs isolated from BMSCs and CA, CA/OP, and OP osteoblasts. *N* = 7. *Significant differences compared to no EVs control Ø with *p* ≤ 0.05. ^#^Tendency to BMSC EVs with *p* = 0.0625. ^§^ Significant differences compared to BMSC-EVs with *p* ≤ 0.05. ^§^ (Red) = significant differences to CA/OP EVs with *p* ≤ 0.05.

Next, we investigated the impact of naïve BMSC-, CA-, CA/ OP-, and OP-derived EVs on osteogenic gene expression in BMSCs (Figure 7A). The gene *BGLAP* is a marker for the late osteogenic differentiation stage and codes for a protein that binds to calcium and hydroxyapatite. *BGLAP* expression revealed no difference in BMSC cultures after stimulation with naïve BMSC, CA, CA/OP, and OP EVs compared to the No EV-control. However, *BGLAP* expression was significantly decreased in the OP EV compared to CA/OP EV-treated BMSC group. *COL1A1* was used as a marker for early osteogenic differentiation. Stimulation with OP EVs significantly decreased *COL1A1* expression compared to the No EV-control. No differences were observed in the other groups. *RUNX2*, a crucial osteogenic transcription factor, was not affected in all groups. *ALP* was used as a marker gene for bone formation processes. Expression was reduced significantly in BMSCs after stimulation with OP EVs and by trend after stimulation with CA- and CA/OP EVs compared to the No EV-control. Naïve BMSC-derived EVs had no effect.

### Effects of BMSC-, CA-, CA/ OP-, and OP-Derived EVs on Osteogenic Differentiation Capacity of BMSCs

ALP enzyme activity was determined as a marker for bone formation activity. Treatment with CA, OP, and CA/OP EVs resulted in a significant decrease in ALP activity compared to the No EV-control, BMSC EV-treated cells remained unaffected. Further, stimulation with CA/OP and CA EVs decreased ALP enzyme activity compared to BMSC EV-treated group (Figure 7B).

To analyze the effect of the different EV groups on calcium deposition ability in osteogenic differentiated BMSCs, Alizarin Red staining was quantified. Stimulation of BMSCs with CA, OP, CA/OP, and BMSC EVs revealed no effect on extracellular calcium deposition compared to the No EV-control, but stimulation with CA EVs reduced calcium deposition compared to BMSC EV-treated cells (Figure 7C).

## Discussion

Already in 2001, Wakitani et al. (2002) reported a beneficial use of MSCs for the treatment of knee OA. Since then, the use of MSC-based therapies revealed promising effects in OA therapy (Orozco et al., 2013; Davatchi et al., 2016; Harrell et al., 2019) and provides a promising approach for the treatment of OP (Paspaliaris and Kolios, 2019; Macías et al., 2020). Nowadays, the therapeutic effect of MSCs had in part been assigned to secreted factors encapsulated in extracellular vesicles, i.e., EVs. The use of EVs as therapeutic drugs has several advantages compared to cell-based strategies as higher safety profile or lower immunogenicity (Gowen et al., 2020) making them an ideal treatment option, also for OA and OP (Cosenza et al., 2017; Zhu et al., 2017; Yang et al., 2020). Most of the present studies report effects of MSC-derived EVs on the respective target cell or disease situation. However, less is known about the effects of EVs derived from already pathologically altered cells. These EVs presumably act different to MSC EVs and might possibly influence treatment outcome due to changes in their cargo, which then exerts different biological effects (Ni et al., 2020). Therefore, we compared the effects of EVs isolated from osteoblasts of patients with CA, OP, or a combination of both (CA/OP) with the effects of BMSC-derived EVs on BMSC metabolism and osteogenic differentiation.

Structural features of trabecular bone explants obtained from the femoral neck from patients with CA, OP, and CA/OP were compared (Figures 1B–E). We found lower trabecular number and higher trabecular separation in bone explants of CA/OP compared to CA and OP patients, pointing to severely impaired structural bone properties when both pathologies are present. Interestingly, no differences regarding these parameters were observed between bone explants of CA and OP patients. Similar to our results, Zhang et al. (2010) did not observe differences in trabecular number and separation between CA and OP biopsies, whereas trabecular thickness was reduced in OP bone explants. The different sample locations—subchondral trabecular bone in their study vs. trabecular bone of a femoral neck disc in our study—might account for this difference. Contrary, Blain et al. described a 50% reduction in trabecular bone volume of OP- compared to OA bone explants from the femoral neck (Blain et al., 2008). However, the origin of the trabecular neck explant with respect to the orientation of the bone biopsy (superior, inferior, posterior, anterior) seems to be important. Trabecular thickness, number and separation were comparable in OA and OP samples of the superior quadrant of the femoral neck, whereas differences were observed when comparing samples of the inferior, anterior, and posterior quadrant (Rubinacci et al., 2012).

Cell growth during outgrowth from bone explants and after thawing was not affected by the different pathologies (Figure 2). In contrast, human OP osteoblasts grew slower compared to OA osteoblasts in a study by Giner et al. (2009). In their study, osteoblasts were isolated by rinsing trabecular bone pieces with PBS and culture of the obtained cells, consisting most probably of precursor cells, lining cells, and osteoblasts from remodeling sites. These cells might be influenced by OP- or OA-specific factors from the surrounding microenvironment, leading to fast but not permanent changes. Osteoblasts in our study grew out of bone explants and, hence, might show rather permanent changes according to the pathologies. Like us, they found similar growth rates when reseeding the cells.

Our data demonstrate that cell viability and the amount of Alizarin Red dye, bound to the extracellular matrix (ECM), increased over time in CA/OP osteoblast ECM, pointing to an increase in osteogenic activity in these cells during culture time, unlike for CA and OP osteoblasts (Figures 3A,B). However, the amount of incorporated Alizarin Red dye into the ECM of CA osteoblasts on days 14–35 was already comparable to the amount in CA/OP osteoblast ECM on day 35. An increasing ALP activity from day 14 to 35, a slight although not significant increase in calcium deposition and a consistently good viability in CA osteoblast cultures let assume a well-functioning osteogenic cell metabolism (Figure 3C). Cell viability increased in OP osteoblasts from days 14 to 35, whereas ALP activity was lower in OP compared to CA osteoblasts (Figures 3A,C). This would suggest a viable growth rate with lower osteogenic cell metabolism in OP osteoblasts under culture conditions. This is in line with a study analyzing bone tissue extracts obtained from normal, OA, and OP patients (Mansell et al., 1997). Elevated ALP levels were present in bone tissue extracts from OA patients, whereas similar levels were measured in samples from unaffected donors and OP patients. Taking together these data let assume, that disease pathologies exert slightly different effects on osteoblast metabolism and ECM production, however, typical markers of an osteogenic cell metabolism were expressed in all groups.

We verified EV isolation from the supernatants of our BMSC and osteoblast cultures using the EV membrane markers CD9 and CD81 (Figure 5C), that have been described as enriched in the membrane of EVs, including exosomes (Andreu and Yáñez-Mó, 2014; Nakamura et al., 2015). Shape and size of the isolated EVs in this study (Figures 5A,B) resembled the typical shape already described by others using TEM and SEM microscopy (Nojehdehi et al., 2018; Takeuchi et al., 2019). Using PKH-26, we demonstrated the uptake of EVs by BMSCs under culture conditions (Figure 5D), which is in line with studies by Ono et al. (2014) and Shabbir et al. (2015).

Positive effects had been reported in the literature for BMSC EVs on parameters regarding cell metabolism. Therefore, we used naïve BMSC EVs to control for CA, OP, and CA/OP EV-mediated effects. We did not observe changes in BMSC proliferation neither after stimulation with BMSC-derived EVs, nor with CA, OP, and CA/OP-derived EVs (Figure 6A). Application of 1 × 10^11^ particles/ml of EVs from synovial-derived MSCs stimulated BMSC proliferation (Guo et al., 2016) and, 25 μg/ml EVs isolated from adipose-derived stem cells also enhanced BMSC proliferation (Li et al., 2018). In our study, EVs were isolated from cells of the same origin (BMSCs of the same donors). In addition, we used 10 μg/ml EVs and proliferation was analyzed over a time period of 24 h in our study, whereas the other studies applied longer observation time points until days 7 (Guo et al., 2016) and 8 (Li et al., 2018). From another perspective, the results support the suggestion that EV cargo is highly cell specific (Valadi et al., 2007; Conde-Vancells et al., 2008; Simpson et al., 2008; Mathivanan et al., 2010; Wu et al., 2015) and might differ between synovial-, adipose-, and bone marrow-derived MSC-EVs. EV content might even differ with respect to donor age of BMSCs, demonstrated by a study with rats, where EVs derived from BMSCs of 2 weeks old rats enhanced proliferation of BMSCs isolated from 15 months old rats (Jia et al., 2020).

We observed reduced viability after stimulation with CA and CA/OP EVs (Figure 6B). The content of these EVs derived from osteoblasts with a CA background, seems to have a negative influence on BMSC viability and might also influence proliferation at later time points. In addition, apoptosis rate was increased in BMSC cultures stimulated with CA and CA/OP EVs, further confirming the catabolic effect of these EVs on BMSC metabolism (Figures 6C,D). Likewise to our study, EVs derived from the synovia of OA patients significantly reduced cell survival of articular chondrocytes (Kolhe et al., 2017) and were able to push inflammatory processes and cartilage degeneration (Domenis et al., 2017), further emphasizing the detrimental effects of EVs derived from OA affected tissues. Analysis of EV cargo in the study of Kolhe et al. revealed altered miRNA expression in OA and non-OA synovia-derived EVs/exosomes, which might account for the catabolic effects. Another important finding of this study was the differently regulated miRNA content in male and female OA EVs compared to non-OA with differently regulated miRNAs in female synovial-derived OA EVs that were responsive to estrogen signaling (Kolhe et al., 2017). Changes in estrogen signaling play a major role in progression of postmenopausal osteoporosis (Raisz, 2005; Weitzmann and Pacifici, 2006). According to literature and our data, we hypothesize differences in cargo of OP- compared to OA-derived EVs. This might in part explain the effects in our studies, as OP EVs did not compromise BMSC proliferation and viability. Nevertheless, stimulation with OP EVs increased apoptosis rate in BMSC cultures similar to CA and CA/OP EVs. The increase of osteoblast and osteocyte death in osteoporotic patients is a known problem (Manolagas, 1998) and seems to be reflected in the cargo of OP-osteoblast derived EVs. In addition, we assume, that the EV content reflects the increase in bone remodeling, that had been reported under osteoporotic conditions (Jilka, 1998; Weinstein and Manolagas, 2000). These changes seem to increase both osteoblast and osteoclast numbers and increase the production of factors, e.g., cytokines, which are included in the EV cargo. We suggest, that EVs of osteoporotic osteoblasts are partly packed with viability promoting factors, reflecting the increase in bone remodeling and the increase in need for osteoblasts. However, estrogen deficiency results in a considerable increase in osteoblast death, due to a faster life cycle and premature aging of osteoblasts (Weinstein and Manolagas, 2000; Masi, 2008), which also seems to be reflected in the EV content.

Another influence on OP EV content might emerge by the femoral neck fracture in the osteoporotic patients. Fracture healing involves a variety of processes throughout all healing phases, from the induction phase to the remodeling phase. Each phase involves a considerable number of different cell types and a variety of different processes. Some of the processes during fracture healing include inflammation, cell migration, differentiation, matrix formation, and resorption. Each process is highly regulated and EVs are most probably involved in the cross-talk between the cells. This can be assumed from literature, demonstrating the positive impact of MSC EVs on fracture healing and bone regeneration (Tan et al., 2020; Zhang et al., 2020). The study of Brady et al. (2018) confirms our suggestion, it reported that shared as well as unique proteins were identified in serum-derived exosomes of dogs with a traumatic bone fracture in comparison to normal dogs.

In accordance with this assumption, Sun et al. (2016) demonstrated increased miR-214 levels in serum-derived EVs of osteoporotic patients. A more detailed analysis of the molecular content of serum-derived osteoporotic EVs revealed a remarkable number of differently expressed proteins, that play a pivotal role in the control of cell differentiation, cell growth, or maintenance, and bone formation processes (Xie et al., 2018). In addition, the authors described decreased ALP levels in the hFOB 1.19 osteoblastic cell line after stimulation with serum-derived osteoporotic EVs. These data support our findings demonstrating that OP EVs reduced ALP gene expression and ALP activity in osteogenic differentiated BMSC cultures (Figures 7A,B).

Stimulation with OP, CA, and CA/OP EVs had comparable effects on *ALP* gene expression and ALP enzyme activity in BMSC cultures (Figures 7A,B). ALP enzyme activity was described as an important mechanism for bone matrix mineralization and deficiencies in ALP activity result in impaired calcium deposition (Anderson et al., 2005; Millán, 2013). However, only CA EVs stimulated BMSC cultures showed reduced Alizarin Red staining (Figure 7C). CA EVs seem to inhibit calcium deposition during osteogenic differentiation of BMSC cultures, whereas this effect was not observed after stimulation with OP and CA/OP EVs. We assume that CA EVs are packed differently compared to OP and CA/OP EVs, thereby affecting deposition of inorganic components during matrix mineralization. In contrast, EVs derived from OP osteoblasts might affect organic components of bone matrix, as we found reduced *COL1A1* gene expression in OP EV stimulated BMSC cultures.

In summary, these results demonstrate a different effect of CA and OP pathologies on osteogenic cell metabolism of osteoblasts. The combined background of both pathologies confers viability comparable to the OP background whereas markers of matrix formation were affected different in single and combined pathologies. According to our data, these differences are not always reflected in their EV cargo. Regarding effects on BMSC metabolism and osteogenic differentiation, CA/OP EVs closely mimic the effects of CA EVs whereas OP EVs exert partly different effects.

## Conclusion

In conclusion, EVs of pathologically altered cells with an OA and OP or combined background differently influence BMSC metabolism and osteogenic markers with respect to bone formation. Hence, the tissue- or cellular microenvironment should not be ignored as it might counteract the desired effects of therapeutically administered EVs. In addition, we suggest that cell origin and donor age are important factors, which should be considered when choosing the cellular source of EVs for therapeutic use.

## Data Availability Statement

The raw data supporting the conclusions of this article will be made available by the authors, without undue reservation.

## Ethics Statement

The studies involving human participants were reviewed and approved by Ethikkommission University of Regensburg, Nos. 14-101-0189 and 18-1109-101, e-mail: ethikkommission@klinik.uni.regensburg.de. The patients/participants provided their written informed consent to participate in this study.

## Author Contributions

TN and CL performed the experiments. CL and SL worked out the exosome isolation and characterization. BC provided the patient samples. CB, MF, and MH contributed to the exosome characterization and verification (TEM, SEM analysis). TN, SS, and SG developed the theory and planned the experiments. TN, CL, and SG wrote the manuscript. All authors discussed the results and commented on the manuscript.

## Conflict of Interest

The authors declare that the research was conducted in the absence of any commercial or financial relationships that could be construed as a potential conflict of interest.

## Supplementary Material

Supplementary Figure 1 Collection **(A)** and isolation **(B)** procedures of EVs from the culture supernatant of BMSCs and CA, CA/OP, and OP osteoblasts (OB) culture flask, reaction tubes, and centrifuge by Servier Medical Art (https://smart.servier.com/; CC BY 3.0).

Supplementary Figure 2 Stimulation of BMSC cultures with naive BMSC-, CA-, CA/ OP-, and OP-derived EVs. BMSCs were thawed and cultured in growth medium until 80–90% confluency. Cells were harvested and seeded in the appropriate well-plates and cultured in growth medium (BrdU, Caspase 3/7, and WST-1 assay), or osteogenic medium (ALP, Alizarin Red assay, and gene expression analysis). Respective stimulation time points are shown in the image culture flasks, plates, and pipettes by Servier Medical Art (https://smart.servier.com/; CC BY 3.0).

Supplementary Figure 3 Ponceau red images of Western Blot membranes used for EV verification by incubation with CD9 (left image) and CD81 (right image) antibodies.
