# Supplementary figures and images for: Influence of Extracellular Vesicles Isolated From Osteoblasts of Patients With Cox-Arthrosis and/or Osteoporosis on Metabolism and Osteogenic Differentiation of BMSCs
Source: Front Bioeng Biotechnol. 2020 Dec 23;8:615520. doi: 10.3389/fbioe.2020.615520 (PMC7785908; doi:10.3389/fbioe.2020.615520)

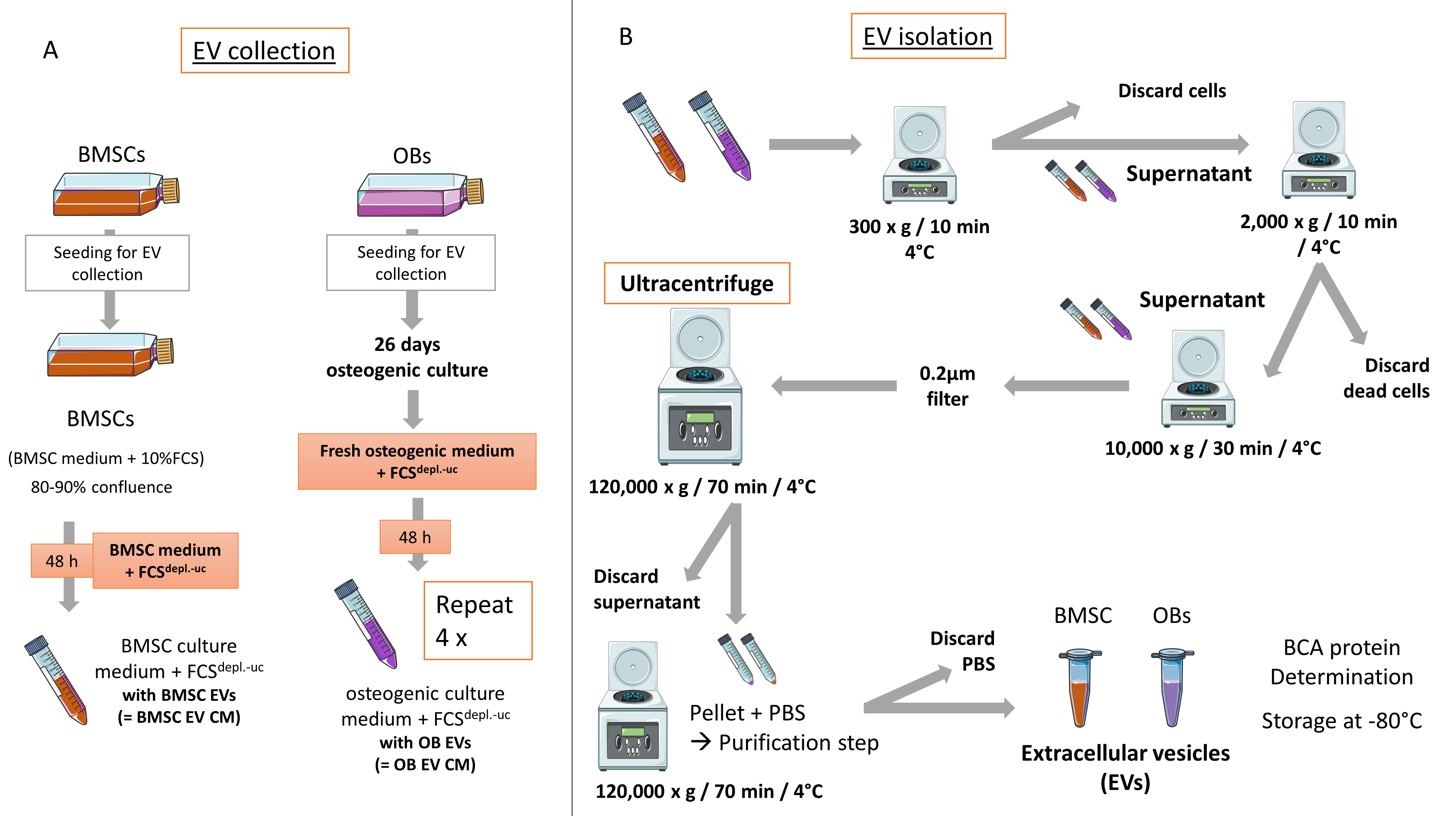

Supplement: Supplementary Figure 1 — Collection (A) and isolation (B) procedures of EVs from the culture supernatant of BMSCs and CA, CA/OP, and OP osteoblasts (OB) culture flask, reaction tubes, and centrifuge by Servier Medical Art (https://smart.servier.com/; CC BY 3.0). [file Image_1.JPEG]

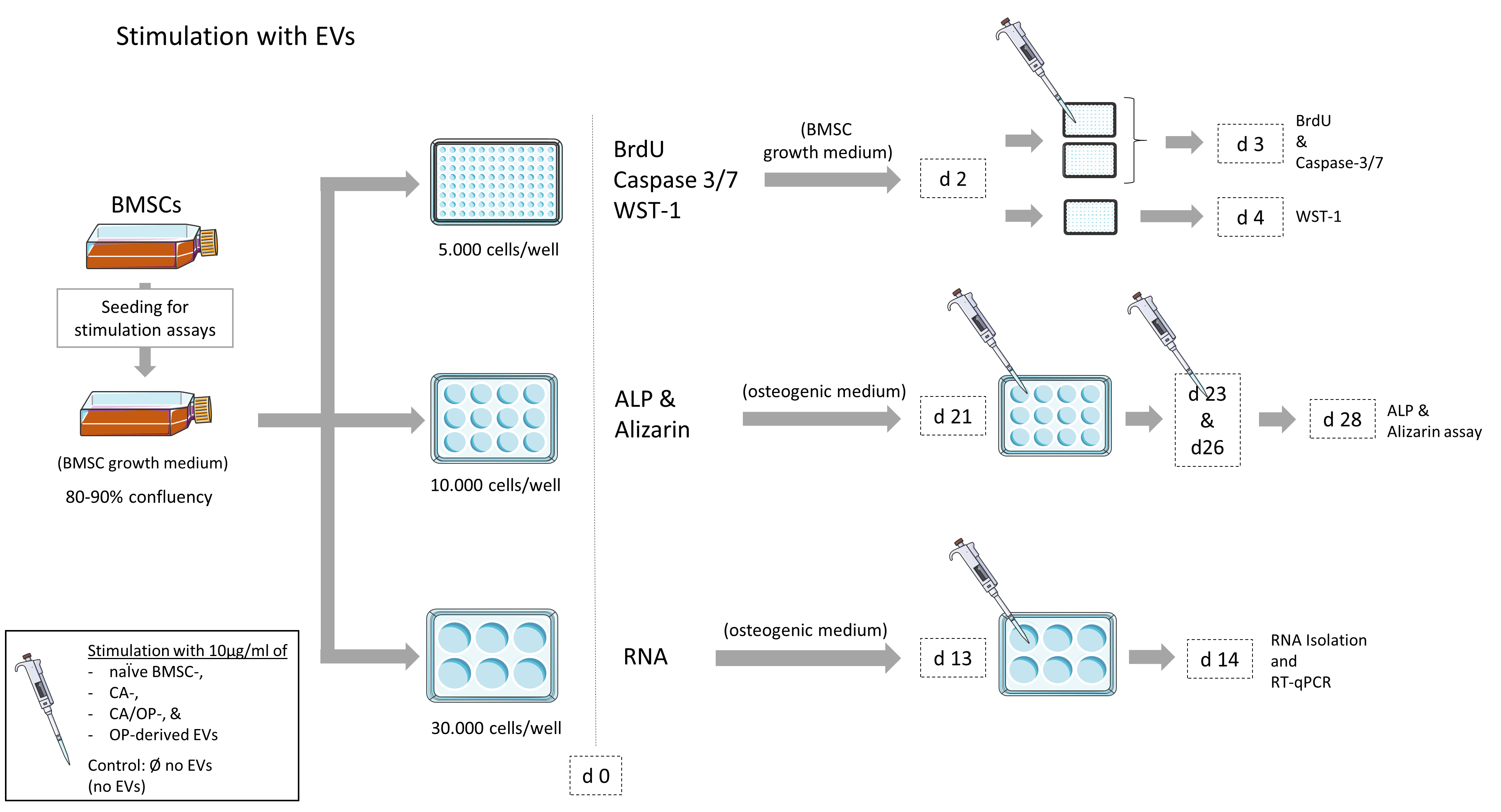

Supplement: Supplementary Figure 2 — Stimulation of BMSC cultures with naive BMSC-, CA-, CA/ OP-, and OP-derived EVs. BMSCs were thawed and cultured in growth medium until 80–90% confluency. Cells were harvested and seeded in the appropriate well-plates and cultured in growth medium (BrdU, Caspase 3/7, and WST-1 assay), or osteogenic medium (ALP, Alizarin Red assay, and gene expression analysis). Respective stimulation time points are shown in the image culture flasks, plates, and pipettes by Servier Medical Art (https://smart.servier.com/; CC BY 3.0). [file Image_2.JPEG]

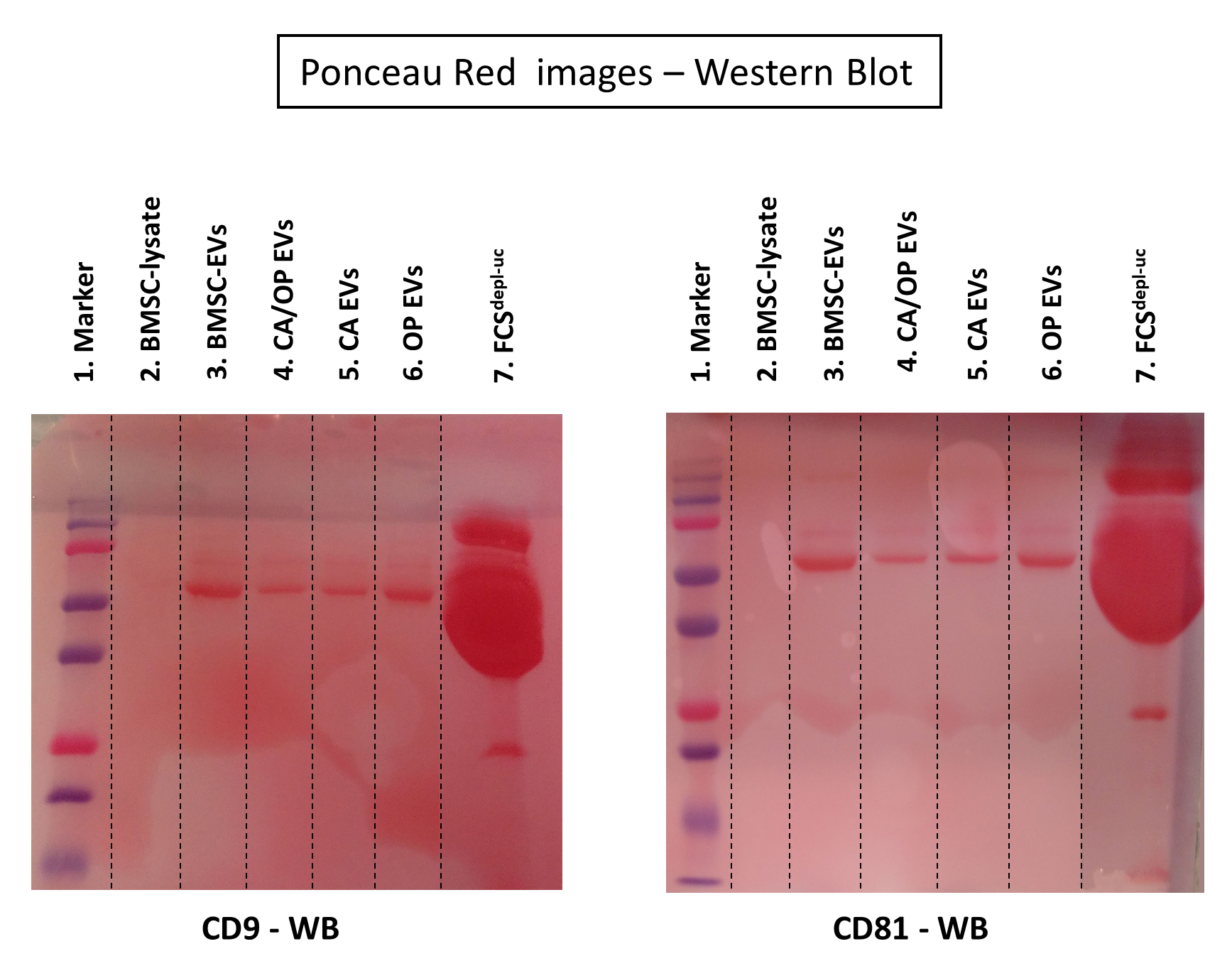

Supplement: Supplementary Figure 3 — Ponceau red images of Western Blot membranes used for EV verification by incubation with CD9 (left image) and CD81 (right image) antibodies. [file Image_3.JPEG]
